# Impact of stress hyperglycemia ratio on incidence of in-hospital cardiogenic shock in patients with ST-elevation myocardial infarction: a prospective, multicenter study

**DOI:** 10.3389/fendo.2025.1677084

**Published:** 2025-12-12

**Authors:** Shan Wang, You Zhang, Wei Yang, Xianpei Wang, Zhongyu Zhu, Datun Qi, Jing Zhang, Jian Zhang, Chuanyu Gao

**Affiliations:** 1 Department of Cardiology, Central China Fuwai Hospital, Central China Fuwai Hospital of Zhengzhou University, Heart Center of Henan Provincial People’s Hospital, Zhengzhou, Henan, China; 2 Henan Institute of Cardiovascular Epidemiology, Zhengzhou, Henan, China; 3 Henan Key Laboratory of Coronary Heart Disease Control & Prevention, Central China Fuwai Hospital, Zhengzhou, Henan, China; 4 State Key Laboratory of Cardiovascular Disease, Heart Failure Center, National Center for Cardiovascular Diseases, Fuwai Hospital, Chinese Academy of Medical Sciences and Peking Union Medical College, Beijing, China

**Keywords:** stress hyperglycemia ratio, ST-elevation myocardial infarction, in-hospital cardiogenic shock, diabetes mellitus, multicenter study

## Abstract

**Background:**

The stress hyperglycemia ratio (SHR) has emerged as a valuable prognostic indicator in patients with ST-elevation myocardial infarction (STEMI). Nevertheless, the impact of SHR on the incidence of in-hospital cardiogenic shock (IHCS) remains insufficiently explored in patients with STEMI. This study aimed to investigate the association between SHR and IHCS incidence while assessing its additional predictive value beyond established risk scores.

**Methods:**

Data were derived from a prospective, multicenter registry and included 1776 patients with STEMI. Patients were grouped by the optimal cutoff value of SHR. The primary endpoint was the incidence of IHCS. Generalized linear mixed models, restricted cubic splines (RCS), ROC curves, and decision curve analysis (DCA) were utilized, with feature importance assessing its importance.

**Results:**

The IHCS incidence was notably higher in patients with elevated SHR. RCS analysis indicated a significant dose-response relationship between increasing SHR and IHCS risk (*P-Nonlinear* > 0.05). SHR was independently associated with IHCS in the overall population (*OR:* 2.19, *95% C*I: 1.20-4.00) and in patients without diabetes (*OR:* 2.20, *95% CI:* 1.11-4.35). Incorporating SHR into established risk scores significantly improved predictive accuracy and net clinical benefit. SHR was comparably important in predicting IHCS compared to established risk factors.

**Conclusions:**

Elevated SHR is a valuable predictor and manifests additional predictive value beyond established risk scores in predicting IHCS in patients with STEMI, especially among patients without diabetes.

**Clinical Trial Registration:**

ClinicalTrials.gov, identifier NCT02641262.

## 1 Introduction

Cardiogenic shock (CS) is a life-threatening complication that indicates a grave prognosis of ST-elevation myocardial infarction (STEMI) despite the widespread implementation of early coronary reperfusion therapy (1–3). Notably, the majority of STEMI patients develop CS during the index hospitalization, and the in-hospital CS (IHCS) is associated with significantly worse outcomes (1, 2, 4). Furthermore, previous clinical trials demonstrated minimal prognostic improvement from mechanical and pharmacological therapies beyond early revascularization (5, 6), which implies that adjunctive therapies may primarily benefit patients at high risk for CS and emphasizes the importance of early risk assessment in making adequate therapeutic decisions and management. Although predictors and scores for mortality in patients already developed CS have been constructed (7, 8), they fail to provide information to assist management and therefore improve clinical outcomes, which highlights the need to identify novel and reliable predictive markers for the occurrence of IHCS and to enhance the accuracy of commonly used scoring systems to improve the prognosis of STEMI patients.

Admission blood glucose (ABG) is a well-established prognostic indicator for STEMI patients and is applied for early prediction of CS (9, 10); nevertheless, ABG reflects both acute stress-induced hyperglycemia (SIH) and chronic glycemic levels, which might limit its efficacy to identify the genuine acute glycemic rise and diminish its prognostic utility, especially in STEMI patients with diabetes mellitus (DM). The stress hyperglycemia ratio (SHR), defined as the ratio of glucose and glycosylated hemoglobin (HbA1c) at admission, was devised to normalize the critical illness-related blood glucose increase on top of the background glycemic status (11). The prognostic value of SHR has been demonstrated across diverse cardiovascular conditions (12, 13). Its association with short- and long-term prognosis of STEMI patients (14, 15), as well as its additional predictive value for in-hospital cardiac arrest (16), heart failure (17), pulmonary infection (18), and new-onset atrial fibrillation (19), have been illustrated. To the best of our knowledge, previous studies on SHR reported CS as part of a composite outcome (20) or assessed its prognostic impact in patients who developed CS (21, 22). The specific relationship between SHR and the incidence of IHCS in STEMI patients has not been fully evaluated, particularly across STEMI patients with different glucose metabolism statuses.

Accordingly, we aimed to investigate the association between SHR and the occurrence of IHCS, as well as the additive effect of SHR on the predictive value of the established classic scoring systems in STEMI patients with and without DM.

## 2 Methods

### 2.1 Study design and population

This study is a *post-hoc* analysis of the *Henan STEMI registry (NCT 02641262)*, which was a multicenter, prospective, observational study to investigate the clinical characteristics, management, and prognosis of STEMI patients (23). Patients were eligible for the registry if they presented with STEMI and were admitted within 30 days of symptom onset. STEMI was defined in accordance with the third universal definition of myocardial infarction (2012), specifically as persistent ST-segment elevation (≥0.1 mV at J points) in two or more contiguous leads or new onset of left bundle branch block. Furthermore, according to the classification of myocardial infarction, patients with types 1, 2, 3, 4b, and 4c of myocardial infarction were eligible. The eligible patients with STEMI were consecutively enrolled from 66 hospitals ***(*****Supplementary Table 1*****)*** in central China. The registry was conducted in accordance with the principles of the Declaration of Helsinki and approved by the Ethics Committee of Henan Provincial People’s Hospital. Due to the life-threatening nature of STEMI, and because all the treatments applied to participants followed relevant guidelines with no additional intervention, waiver of informed consent had been approved by the ethics committee, and the other participating institutes were covered by central ethics approval.

From September 2016 to August 2018, the registry enrolled a total of 5063 subjects diagnosed with STEMI. Patients who met the following criteria were excluded: (1) ABG deficiency within 24 hours after admission; (2) HbA1c deficiency during the index hospitalization; (3) those complicated with out-of-hospital CS; (4) those without serum creatinine data during the index hospitalization. As a result, 1776 STEMI patients were included in this analysis. Patients were grouped according to the optimal cutoff value of SHR for predicting IHCS determined by the receiver operating characteristic (ROC) curve analysis in the total population and a subset of DM and non-DM patients (**Supplementary Figure 1**).

### 2.2 Data collection and definitions

The demographics, medical histories, cardiovascular risk factors, clinical characteristics at admission, reperfusion therapy, medications during hospitalization, laboratory results, and clinical events were prospectively collected using a web-based and password-protected data collection platform. This platform was capable of real-time automatic logic and range check on the completeness and validity of the data and automatically reminded investigators of follow-ups. The enrolled patients were assigned a unique ID identified through the patients’ ID numbers to avoid duplicate input and for data query and revision. Data were collected and submitted by investigators of each participating center. All the investigators received a detailed training program on protocol, data collection, and software system before the registry started, and the investigator meetings were annually held to intensify the effect of the training, and a WeChat group was established to facilitate communication and solve problems related to the registry. Additionally, the registry study group regularly checked the data quality and sent queries for missing or illogical data to participating sites for review and revision. We also verified consecutive enrollment and audited for accuracy against medical records for onsite quality control. Ultimately, a total of 53.84% of reported cases were audited (23).

ABG was defined as the first measured random blood glucose within 24 hours after hospital admission. SHR was calculated using this formula: ABG (mmol/L)/(1.59*HbA1c (%)-2.59) (11). Diabetes mellitus was defined as having a history of diabetes mellitus or an HbA1c level ≥6.5% at admission. Out-of-hospital cardiac arrest (OHCA) was defined as presenting with a cardiac arrest (CA) diagnosis before hospital admission, and CA was identified by the cardiologist at each participating center in line with guidelines (24). Hypertension was defined as having a history of hypertension or receiving antihypertensive therapy. Dyslipidemia was defined according to the guidelines for the prevention and treatment of dyslipidemia in Chinese adults (revised in 2016) (25). Current smoking was defined as smoking within the preceding year. Stroke was defined as a history of cerebral hemorrhage or ischemic stroke. The anterior myocardial infarction was determined by an electrocardiogram. Malignant arrhythmia was defined as sinus arrest, ventricular tachycardia, ventricular flutter, ventricular fibrillation, and atrial ventricular block (II-degree type 2 and III degrees). The Thrombolysis in Myocardial Infarction (TIMI) risk score and Global Registry of Acute Coronary Events (GRACE) score were calculated according to previous studies.

### 2.3 Endpoints

The primary endpoint was the incidence of IHCS during hospitalization. The diagnosis of CS was identified by the cardiologist at each participating center in accordance with the current guidelines (26), and all the CS diagnoses were adjudicated by the senior cardiologist of the registry study group based on the clinical characteristics and laboratory results in the medical records.

### 2.4 Statistical analyses

The demographic, clinical characteristics, treatment therapy, and laboratory results were compared between the higher and the lower SHR group, as well as patients who developed IHCS and those without IHCS. The categorical variables were presented as numbers and percentages, and *Chi-square* or *Fisher’s* exact tests were used for comparisons as appropriate, whereas continuous variables were reported as means and standard deviation (SD) or median and interquartile range (IQR), *and t-test* or Mann-Whitney *U* test was used as appropriate.

The ROC curve was applied to determine the optimal cutoff value of SHR for predicting IHCS in the total population and the subsets of DM and non-DM patients. The generalized linear mixed model, which accounted for clustering of patients within hospitals, was performed to explore the association between SHR and the occurrence of IHCS, with the lower SHR group as reference, and results were reported as odds ratios (OR) with associated 95% confidence intervals (CIs). In this analysis, model 1 only accounted for clustering of patients within hospitals, model 2 was adjusted for demographic characteristics (age, gender, hospital grade and weight) on model 1 basis, model 3 was adjusted for risk factors and medical history (hypertension, diabetes, dyslipidemia, current smoker, and stroke) on model 2 basis, model 4 was adjusted for clinical characteristic on admission (Killip class (II/III vs I), malignant arrhythmia, LBBB, atrial fibrillation or flutter, SBP, heart rate and serum creatinine) on model 3 basis, model 5 adjusted for medical therapy (reperfusion therapy (thrombolysis or PCI vs none), onset-to-FMC time) on model 4 basis, model 6 was adjusted for medications (aspirin, P2Y12 antagonists, statin, β-blocker and ACEI)/ARB) on model 5 basis, model 7 was adjusted for TIMI risk score on model 1 basis, and model 8 was adjusted for GRACE score on model 1 basis. Additionally, the association between SHR (as a continuous variable) with the occurrence of IHCS was modelled using restricted cubic splines (RCS) analysis.

The area under the curve (AUC) was calculated and utilized to quantify the predictive ability of SHR as well as the TIMI risk score and GRACE score for IHCS. AUC comparisons of SHR beyond the TIMI risk score and GRACE score were assessed using DeLong’s test. Additionally, the integrated discrimination improvement (IDI) and category-free net reclassification improvement (NRI) were applied to evaluate the incremental effect of SHR in risk predictions quantified on top of classic scores. Decision curve analysis (DCA) was conducted to determine the incremental clinical usefulness of SHR by quantifying the net benefits at different threshold probabilities. We further employed permutation feature importance analysis to determine the feature importance of higher SHR and the established classic risk predictors that were included in the TIMI risk score and GRACE score on the occurrence of IHCS. Feature importance was calculated as the mean feature importance obtained from a large number of independent random shuffles in a random forest model. Internal validation with 1,000 bootstrap resamples was used to estimate the accuracy of the prediction models and to reduce overfitting bias. Finally, we performed subgroup analyses to assess factors associated with the occurrence of IHCS, the prognosis of STEMI patients, and factors potentially influencing SHR’s predictive performance, using the generalized linear mixed model that accounted for clustering of patients within hospitals and adjusted for DM status.

A two-sided *P* value <0.05 was considered statistically significant. Statistical analyses were performed using SAS 9.4 (SAS Institute Inc., Cary, NC) and the R package (Version 4.2.1, R Foundation for Statistical Computing, Vienna, Austria).

## 3 Results

### 3.1 Baseline characteristics

A total of 1776 patients with STEMI were included in this analysis, and 373 were identified with DM and 1403 without DM. The median SHR was 0.970. The optimal cutoff value of SHR was 1.295 for the total population, 1.520 for DM patients, and 1.169 for non-DM patients, and STEMI patients were further grouped according to the optimal cutoff value as higher SHR and lower SHR (**Supplementary Figures 1**, **2**). The incidence of IHCS was 4.2% in the total population, and occurred in 9.2% of patients with higher SHR (SHR >= 1.295), which was significantly higher than in patients with lower SHR (SHR < 1.295). Accordingly, 4.4% and 2.4% developed IHCS among DM and non-DM patients, both incidences of IHCS were observed to be higher in patients with higher SHR (SHR >= 1.520 for DM patients, SHR >= 1.169 for non-DM patients) (**Supplementary Figure 3***).*

The baseline characteristics between the higher and lower SHR groups, as well as IHCS and non-IHCS patients, are summarized in **Table 1**. Overall, the mean age of patients was 61.8 years, and 75.7% of the patients were male. The patients with higher SHR and patients complicated with IHCS were older, and presented higher heart rates, ABG, SHR, TIMI risk score, and GRACE score, moreover, the proportion of females, lower weight, previous diabetes, current smokers, Killip class *II/III*, malignant arrhythmia, elevated serum creatinine were significantly higher, nevertheless, those patients showed shorter onset-to-FMC time and were less likely to use ACEI/ARB during hospitalization than their counterpart. In addition, the proportion of patients in secondary hospitals, prior angina, and COPD was higher in higher SHR patients, whereas aspirin, P2Y12 antagonists, and statins were less likely to be used in these patients. Simultaneously, patients with complicated IHCS were more likely to be admitted to tertiary hospitals and presented a higher proportion of prior strokes and lower usage of beta-blockers.

**Table 1:** Baseline demographic and clinical characteristics according to SHR and the occurrence of IHCS.

Variable	Lower SHR (N = 1440)	Higher SHR (N = 336)	*P* value	Non-IHCS (N = 1702)	IHCS (N = 74)	*P* value
Age, years, median (IQR)	62.4 (52.2,69.9)	63.6 (53.0,72.0)	0.012	62.4 (52.2,70.0)	69.7 (61.3,77.2)	<0.001
<65, n (%)	846 (58.8)	178 (53.0)	0.012	1001 (58.8)	23 (31.1)	<0.001
65~74, n (%)	394 (27.4)	90 (26.8)		459 (27.0)	25 (33.8)	
≥75, n (%)	200 (13.9)	68 (20.2)		242 (14.2)	26 (35.1)	
Female, n (%)	325 (22.6)	106 (31.6)	<0.001	402 (23.6)	29 (39.2)	0.002
Tertiary hospital, n (%)	902 (62.6)	188 (56.0)	0.023	1032 (60.6)	58 (78.4)	0.002
Weight< 67Kg	501 (34.8)	154 (45.8)	<0.001	619 (36.4)	36 (48.7)	0.032
Comorbidities and risk factors						
Hypertension, n (%)	616 (42.8)	162 (48.2)	0.071	736 (43.2)	42 (56.8)	0.022
Dyslipidemia, n (%)	884 (61.4)	206 (61.3)	0.979	1044 (61.3)	46 (62.2)	0.887
Diabetes, n (%)	272 (18.9)	101 (30.1)	<0.001	349 (20.5)	24 (32.4)	0.014
Current smoker, n (%)	640 (44.4)	113 (33.6)	<0.001	735 (43.2)	18 (24.3)	0.001
Prior angina, n (%)	158 (11.0)	50 (14.9)	0.045	196 (11.5)	12 (16.2)	0.218
Prior MI, n (%)	83 (5.8)	21 (6.3)	0.733	99 (5.8)	5 (6.8)	0.933
Prior HF, n (%)	12 (0.8)	3 (0.9)	1.000	13 (0.8)	2 (2.7)	0.127
Prior Stroke, n (%)	194 (13.5)	47 (14.0)	0.804	225 (13.2)	16 (21.6)	0.039
Chronic renal insufficiency, n (%)	4 (0.3)	3 (0.9)	0.256	6 (0.4)	1 (1.4)	0.258
Prior COPD, n (%)	21 (1.5)	11 (3.3)	0.024	30 (1.8)	2 (2.7)	0.882
Peripheral arterial disease, n (%)	4 (0.3)	3 (0.9)	0.256	7 (0.4)	0 (0.0)	1.000
Clinical characteristic on admission						
MI symptoms, n (%)						
Typical	1276 (88.6)	294 (87.5)	0.848	1502 (88.3)	68 (91.9)	0.772
Atypical	152 (10.6)	39 (11.6)		185 (10.9)	6 (8.1)	
No symptom	12 (0.8)	3 (0.9)		15 (0.9)	0 (0.0)	
OHCA, n (%)	70 (4.9)	8 (2.4)	0.053	73 (4.3)	5 (6.8)	0.469
Anterior MI, n (%)	820 (56.9)	211 (62.8)	0.057	984 (57.8)	47 (63.5)	0.331
Killip class, n (%)						
I	1179 (81.9)	254 (75.6)	0.009	1384 (81.3)	49 (66.2)	0.001
II / III	261 (18.1)	82 (24.4)		318 (18.7)	25 (33.8)	
Cardiac enzyme elevation, n (%)	1058 (73.5)	257 (76.5)	0.256	1255 (73.7)	60 (81.1)	0.158
Malignant arrhythmia, n (%)	92 (6.4)	36 (10.7)	0.006	118 (6.9)	10 (13.5)	0.032
LBBB on admission, n (%)	16 (1.1)	3 (0.9)	0.956	15 (0.9)	4 (5.4)	0.007
Atrial fibrillation or flutter, n (%)	39 (2.7)	9 (2.7)	0.976	42 (2.5)	6 (8.1)	0.010
SBP, mmHg, median (IQR)	130 (116,146)	130 (112,148)	0.971	130 (116,147)	118 (100,132)	<0.001
SBP < 100 mmHg, n (%)	105 (7.3)	32 (9.5)	0.167	119 (7.0)	18 (24.3)	<0.001
HR, bpm, median (IQR)	75 (66,85)	82 (69,96)	<0.001	76 (66,87)	83 (66,100)	0.006
HR>100 bpm, n (%)	108 (7.5)	57 (16.96)	<0.001	147 (8.64)	18(24.32)	<0.001
Onset-to-FMC, min, median (IQR)	240 (115,841)	198 (115,416)	0.002	233.5 (115,700)	198.5 (120,970)	0.966
Onset-to-FMC>4 hours, n (%)	702 (48.8)	139 (41.4)	0.015	806 (47.4)	35(47.3)	0.992
Reperfusion therapy, n (%)						
PCI	520 (36.1)	122 (36.3)		612 (36.0)	30 (40.5)	0.513
Fibrinolysis	319 (22.2)	85 (25.3)		391 (23.0)	13 (17.6)	
Serum creatinine, umol/L, median (IQR)	69.0 (58.6,80.0)	65.9 (55.0,82.0)	0.135	68.0 (58.0,80.0)	80.5 (60.0,105.0)	<0.001
Serum creatinine>=100umol/L	103 (7.2)	40 (11.9)	0.004	122 (7.2)	21 (28.4)	<0.001
ABG, mmol/L, median (IQR)	6.1 (5.3,7.5)	10.9 (8.7,14.5)	<0.001	6.6 (5.4,8.8)	8.9 (6.7,13.0)	<0.001
HbA1c, %, median (IQR)	6.0 (5.5,7.0)	5.9 (5.3,7.1)	0.012	6.0 (5.5,7.0)	6.3 (5.5,7.7)	0.046
Medicine used during hospitalization						
Aspirin, n (%)	1420 (98.6)	320 (95.2)	<0.001	1670 (98.1)	70 (94.6)	0.092
P2Y12 antagonists, n (%)	1421 (98.7)	324 (96.4)	0.005	1674 (98.4)	71 (96.0)	0.273
Statin, n (%)	1380 (95.8)	313 (93.2)	0.036	1622 (95.3)	71 (96.0)	1.000
Beta-blocker, n (%)	1042 (72.4)	232 (69.1)	0.225	1231 (72.3)	43 (58.1)	0.008
ACEI/ARB, n (%)	836 (58.1)	170 (50.6)	0.013	977 (57.4)	29 (39.2)	0.002
TIMI risk score, median (IQR)	3 (2,5)	4 (2,6)	<0.001	3 (2,5)	6 (4,8)	<0.001
GRACE score, median (IQR)	140 (120,161)	147 (123,173)	<0.001	140 (120,162)	172 (147,193)	<0.001
SHR, median (IQR)	0.90(0.74,1.04)	1.52 (1.38,1.77)	<0.001	0.96 (0.78,1.19)	1.10 (0.89,1.56)	<0.001

Values are presented as median (IQR) or n (%).

STEMI, ST-elevation myocardial infarction; SHR, stress hyperglycemia ratio; IHCS, in-hospital cardiogenic shock; MI, myocardial ischemia; HF, heart failure; COPD, chronic obstructive pulmonary disease; OHCA, out-hospital cardiac arrest; LBBB, left bundle branch block; SBP, systolic blood pressure; HR, heart rate; BPM, beat per minute; FMC, first medical contact; PCI, percutaneous coronary intervention; ABG, admission blood glucose; HbA1c hemoglobin A1c; TC, total cholesterol; LDL-C, low-density lipoprotein cholesterol; P2Y12, clopidogrel or ticagrelor; ACEI, angiotensin-converting enzyme inhibitor; ARB, angiotensin receptor blocker; TIMI, thrombolysis in myocardial infarction; GRACE: Global Registry of Acute coronary events.

### 3.2 Association between SHR and IHCS

**Figure 1:**
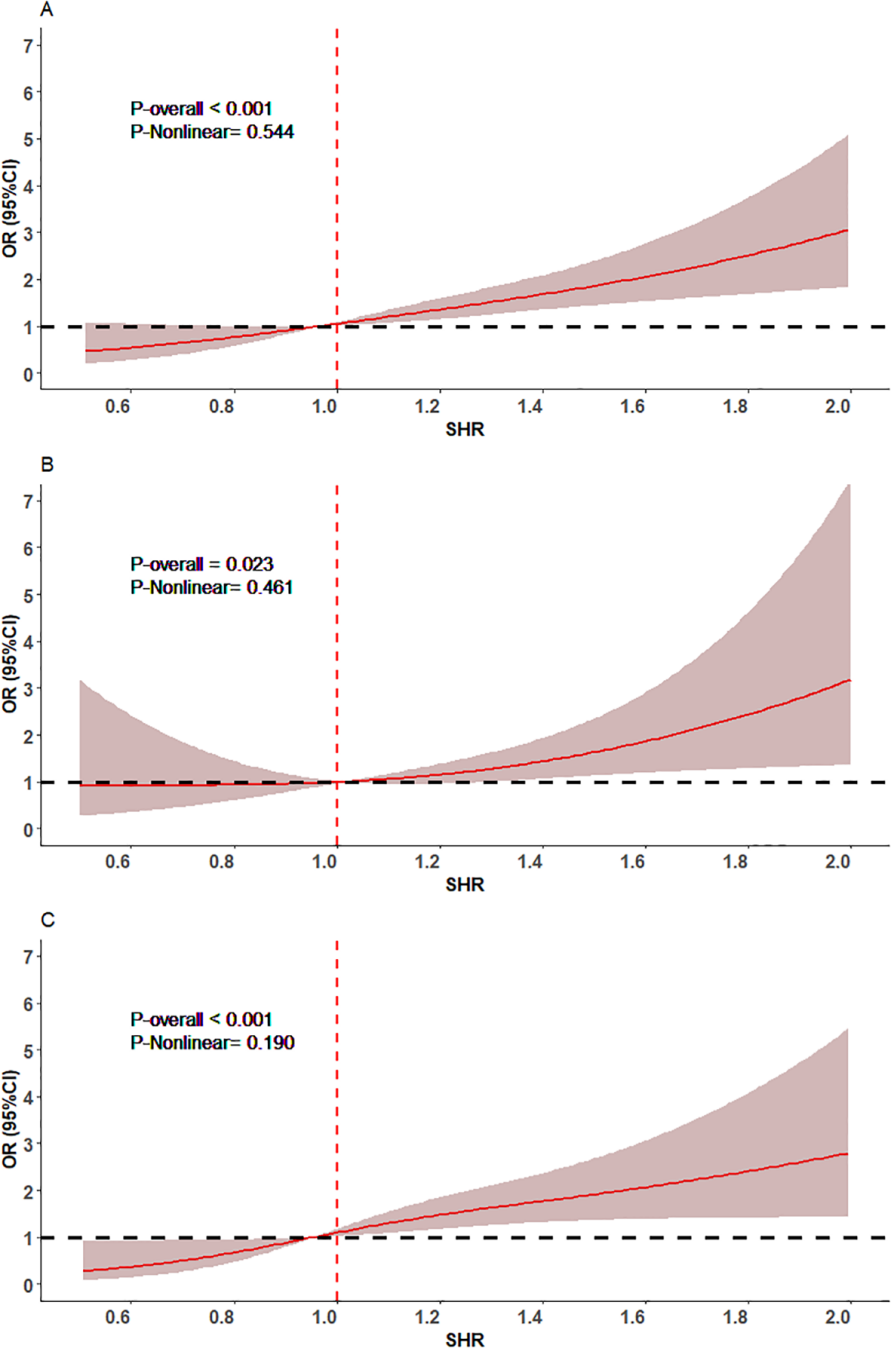
The association of SHR with IHCS measured by restricted cubic spline regression. **(A)** The total patients, **(B)** patients with diabetes, **(C)** patients without diabetes. The baseline (red) line is hazard ratio, and the red shaded area represents the 95% CI.

The RCS analysis indicated a positive linear dose-response relationship between SHR as a continuous variable and IHCS among the total population (*P-Nonlinear* = 0.544) and individuals without DM (*P-Nonlinear* = 0.190), and the OR for the occurrence of IHCS increased significantly with SHR increase (**Figure 1**). Higher SHR was significantly associated with higher risk of IHCS incidence both in the total population (OR: 3.42, 95%CI: 2.03-5.77) and the subsets of non-DM patients (OR: 2.95, 95%CI: 1.59-5.46) after accounting for the clustering of patients within hospitals. What’s more, the adjustments for demographic characteristics, risk factors and medical history, clinical characteristic on admission, medical therapy, and medications used during hospitalization did not fundamentally alter this significant association between SHR and IHCS observed in the unadjusted model both among the total population (OR: 2.19, 95%CI: 1.20-4.00) and patients without DM (OR: 2.20, 95%CI: 1.11-4.35). Additionally, a similar pattern was observed in the further adjustment of the TIMI risk score and GRACE score among the total population and individuals without DM (**Table 2**).

**Table 2:** Univariate and multivariate General mixed model analysis for IHCS according to categorized SHR.

Models	Total patients		DM patients		Non-DM patients	
	OR (95% CI)	*P value*	OR (95% CI)	*P value*	OR (95% CI)	*P value*
Model 1	3.42 (2.03,5.77)	<0.001	5.03 (1.88,13.45)	0.003	2.95 (1.59,5.46)	0.001
Model 2	2.94 (1.72,5.04)	<0.001	3.79 (1.38,10.40)	0.013	2.70 (1.44,5.06)	0.003
Model 3	2.81 (1.63,4.83)	<0.001	3.82 (1.37,10.63)	0.013	2.68 (1.43,5.03)	0.003
Model 4	2.28 (1.27,4.09)	0.007	2.97 (0.87,10.16)	0.079	2.24 (1.15,4.37)	0.020
Model 5	2.24 (1.24,4.04)	0.009	3.15 (0.91,10.90)	0.068	2.16 (1.10,4.23)	0.027
Model 6	2.19 (1.20,4.00)	0.012	2.99 (0.77,11.69)	0.108	2.20 (1.11,4.35)	0.025
Model 7	2.34 (1.35,4.06)	0.004	4.04 (1.34,12.19)	0.016	2.47 (1.32,4.65)	0.006
Model 8	2.52 (1.45,4.36)	0.002	3.95 (1.37,11.41)	0.014	2.55 (1.35,4.80)	0.005

DM, diabetes mellitus; OR, odds ratio. CI, confidence intervals. OR were estimated using general mixed model with the lower SHR as reference and account for clustering of patients within hospitals.

**Model 1,** SHR were included in the model as binary categorical variable (higher SHR and lower SHR) according to the optimal cutoff point.

**Model 2,** on model 1 basis, adjusted for demographic characteristics (age, gender, hospital grade and weight).

**Model 3**, on model 2 basis, adjusted for risk factors and medical history (hypertension, diabetes, dyslipidemia, current smoker, and stroke).

**Model 4**, on model 3 basis, adjusted for clinical characteristic on admission (Killip class (II/III vs I), malignant arrhythmia, LBBB, atrial fibrillation or flutter, SBP, heart rate and serum creatinine).

**Model 5,** on model 4 basis, adjusted for medical therapy (reperfusion therapy (thrombolysis or PCI vs none), onset-to-FMC time).

**Model 6,** on model 5 basis, adjusted for medications (aspirin, P2Y12 antagonists, statin, β-blocker and ACEI)/ ARB).

**Model 7,** on model 1 basis, adjusted for the TIMI risk score.

**Model 8,** on model 1 basis, adjusted for the GRACE score.

In patients with DM, the RCS analysis showed a linear relationship between SHR and IHCS incidence (*P-Nonlinear* = 0.461). Moreover, in the unadjusted model 1 (OR: 5.03, 95%CI: 1.88-13.45), model 2 adjusted for demographic characteristics (OR: 3.79, 95%CI: 1.38-10.40), model 3 adjusted for risk factors and medical history (OR: 3.82, 95%CI: 1.37-10.63), and models adjusted for TIMI risk score (OR: 4.04, 95%CI: 1.34-12.19) and GRACE score (OR: 3.95, 95%CI: 1.37-11.41), higher SHR was an independent predictor of IHCS occurrence. However, the association was not significant in the further adjustments of clinical characteristics on admission *(model 4)*, and medical therapy *(model 5)*, as well as medications used during hospitalization (**Table 2**).

The power of the association analysis between SHR an IHCS was estimated using the estimation procedure for logistic regression in the *PASS* software, under the parameters setting according to the above results (significance level of 0.05, two-tailed, incidence of 4.2%, odds ratio of 3.42), a sample size of 200 participants yielded a power of 0.937. This provided sufficient power to detect the association.

### 3.3 Incremental effect and clinical usefulness of SHR on predicting IHCS

**Figure 2:**
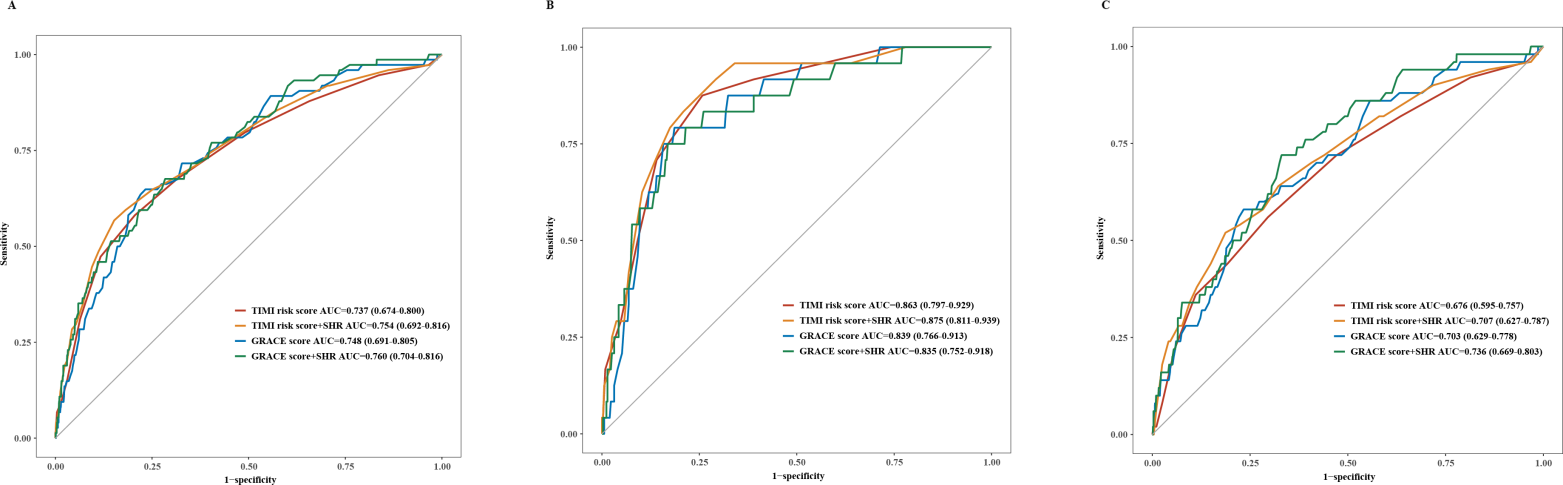
ROC curve of SHR, TIMI risk score and GRACE score for IHCS incidence in STEMI patients. **(A)** ROC curve in the total patients, **(B)** ROC curve in patients with diabetes, **(C)** ROC curve in patients without diabetes. AUC area under curve; CI confidence interval; SHR stress hyperglycemia ratio.

**Figure 3:**
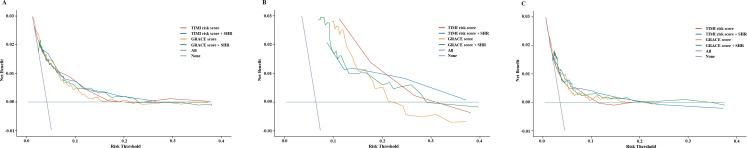
Decision curve analysis (DCA) of SHR, TIMI risk score and GRACE score for IHCS incidence in STEMI patients. **(A)** DCA in the total patients, **(B)** DCA in patients with diabetes **(C)** DCA in patients without diabetes.

The ROC curve analysis demonstrated that SHR had a moderate predictive value for the occurrence of IHCS in the total population, as well as patients with and without DM (**Supplementary Figure 2**). Furthermore, in the total population, adding SHR to the TIMI risk score (AUC: 0.754, 95%CI: 0.692-0.816) or GRACE score (AUC: 0.760, 95%CI: 0.704-0.816) (**Figure 2A**), did not significantly improve the AUC. The results remained consistent after bias-correcting when examined with the internal bootstrap validation method (**Supplementary Table 2**), which indicates a low risk of overfitting or optimism bias, and a stable predicting performance. Whereas, the IDI and NRI were significantly improved after adding SHR to the classic scores, respectively (**Table 3**). Moreover, as the DCA analysis presented in **Figure 3A**, adding SHR to the TIMI risk score resulted in an improvement in the net clinical benefit if the threshold probability was in the interval of 0.12-0.28, and SHR plus GRACE score added more benefit than the GRACE score.

**Table 3:** Comparison of the improvement of discrimination and classification ability of SHR with the TIMI risk score and GRACE score for IHCS in STEMI patients.

Scoring system	AUC difference (95% CI)	P value	NRI (95% CI)	*P* for NRI	IDI (95% CI)	*P* for IDI
Total patients						
TIMI risk score + SHR ^†^	0.017 (-0.008,0.041)	0.176	0.479 (0.252,0.707)	<0.001	0.014 (0.004,0.024)	0.008
GRACE score + SHR ^‡^	0.012 (-0.015,0.038)	0.384	0.479 (0.252,0.707)	<0.001	0.017 (0.006,0.027)	0.002
DM patients						
TIMI risk score + SHR ^†^	0.025 (-0.011,0.061)	0.166	0.564 (0.163,0.965)	0.008	0.033 (-0.011,0.078)	0.139
GRACE score + SHR ^‡^	0.006 (-0.027,0.039)	0.708	0.564 (0.163,0.965)	0.008	0.053 (0.008,0.098)	0.022
Non-DM patients						
TIMI risk score + SHR ^†^	0.031(-0.016,0.078)	0.192	0.490 (0.209,0.771)	<0.001	0.013 (0.004,0.021)	0.005
GRACE score + SHR ^‡^	0.033 (-0.015,0.080)	0.177	0.490 (0.209,0.771)	<0.001	0.012 (0.002,0.021)	0.014

^†^Compared with TIMI risk score; ^‡^Compared with GRACE score. SHR was incorporated in the TIMI risk score and GRACE score as categorical variable (higher SHR or lower SHR).

AUC, area under the curve; CI, confidence intervals; NRI, net reclassification improvement; IDI, integrated discrimination improvement; TIMI, thrombolysis in myocardial infarction; GRACE, global registry of acute coronary events.

Additionally, in patients with DM, the AUC of SHR plus TIMI risk score (AUC: 0.875, 95%CI: 0.811-0.939) and SHR plus GRACE score (AUC: 0.835, 95%CI: 0.752-0.918) were not significantly different from that of TIMI risk score (*P* = 0.166) and GRACE score (*P* = 0.708) (**Figure 2B**, **Table 3**). However, adding SHR resulted in an improvement in NRI compared with the classic scores, and the improvement of IDI compared with the GRACE score was also observed (**Table 3**). The DCA showed that there was no added net benefit compared with classic scores (**Figure 3B**).

In individuals without DM, adding SHR to the TIMI risk score (AUC: 0.707, 95%CI: 0.627-0.787) and GRACE score (AUC: 0.736, 95%CI: 0.669-0.803) demonstrated a non-significant difference in AUC compared to the original scores (**Figure 2C**, **Table 3**). Consistent with the total patients, a significant improvement in NRI and IDI of adding SHR to classic scores was observed (**Table 3**). Moreover, the DCA indicated a clear net benefit of adding SHR to original scores for predicting IHCA and assisting management (**Figure 3C*****).***

The power for detecting the additive predicting effect of SHR in this current study was performed using a sample size algorithm for developing clinical prediction models by the *R* package (pmsampsize). To achieve the expected power of the test (significance level of 0.05, two-tailed, an AUC of 0.750, 2 parameters in the model (GRACE score or TIMI score and SHR in the model), the prevalence of 4.2%, and shrinkage of 0.9), at least 508 participants were required for the sample. The current study provided sufficient power to detect the incremental predictive effect of SHR.

### 3.4 Importance of SHR in predicting IHCS

The permutation feature importance analysis was constructed to assess the relative importance of the 12 established risk factors (older age, lower SBP, elevated heart rate, Killip class II/III, anterior MI or LBBB, medical history (diabetes or HTN or angina), lower weight, treatment delay, elevated creatinine, cardiac arrest at admission, ST-segment deviation, elevated cardiac enzymes/markers, as listed in **Supplementary Table 3**) which were included in the TIMI risk score and GRACE score and elevated SHR for predicting IHCS respectively. The results indicated that the elevated SHR was an important predictor for the prediction of IHCS compared with the established risk factors regardless of glucose metabolism status, and ranked as the fourth important predictor in the total population and patients with DM, and ranked as the second important predictor in patients without DM (**Supplementary Figure 4**).

### 3.5 Subgroup analyses

Subgroup analyses were performed to examine the association between SHR and IHCS incidence in different populations according to age (≥ 65 or < 65 years), gender (female or male), hospital grade (Secondary or tertiary), hypertension (yes or no), dyslipidemia (yes or no), reperfusion therapy (conservative strategy, PCI, or fibrinolysis), onset-to-FMC time (<= 4 or > 4 hours), and anterior MI (yes or no) with accounted for clustering of patients within hospitals and adjusted for DM status.

As shown in **Supplementary Table 4**, the incidence of IHCS ranged from 2.25% to 6.78%, and the OR ranged from 1.03 to 5.51. Higher SHR was significantly associated with IHCS across the subgroups of hospital grade, hypertension, dyslipidemia, reperfusion therapy, and onset-to-FMC time, and the association was consistent between those groups, with no statistically significant interactions observed (all *P* > 0.05). Furthermore, the statistical significance was observed only among males (OR: 4.02, 95%CI: 2.03-7.95) and patients diagnosed with anterior MI (OR: 5.06, 95%CI: 2.63-9.74). Interestingly, although higher SHR was significantly associated with IHCS in both age groups, higher SHR might be a stronger predictor in patients <65 years (OR: 3.19, 95% CI: 1.29-7.88) than in patients >= 65 years (OR: 2.75, 95% CI: 1.44-5.26), with a statistically significant interaction between age groups (*P* for interaction < 0.001).

## 4 Discussion

This prospective, multicenter, population-based registry study revealed a significant association between elevated SHR and IHCS incidence and demonstrated a positive dose-response relationship between SHR and risk of IHCS in STEMI patients, especially in patients without diabetes. Analogously, incorporating SHR into the conventional risk scores indicated an incremental predictive performance, as characterized by significantly increased IDI and NRI. Moreover, the addition of SHR resulted in increased net benefit and could enhance the risk prediction ability of classic risk scores for IHCS. Additionally, the higher SHR manifested comparable importance in predicting the occurrence of IHCS as the established risk factors.

SIH has been reported to be a reliable predictor of adverse outcomes in critically ill patients (27). In STEMI patients, the SIH primarily arises from *β*-cell dysfunction and insulin resistance (28, 29), on the one hand, pancreatic β-cell dysfunction impairs insulin release and reduces proinsulin concentration, while the sympathetic nervous system activation increases glucagon, cortisol, and cytokines, thereby stimulating glucose production through increased gluconeogenesis and glycogenolysis (28). These pathological process further promotes occurrence of insulin resistance through multiple mechanisms, including mobilization of activation of serine/threonine kinases that interfere with insulin signaling, and the dysregulation of both the sympathetic nervous and renin-angiotensin-aldosterone systems, and resulting in excessive stimulation of adrenergic and angiotensin II receptors (30). Crucially, the diminished insulin secretion cannot adequately counteract the hyperglycemic effects of these counter-regulatory hormones and cytokines (29). Collectively, stress hyperglycemia initiates a cascade of detrimental consequences, including triggering inflammation and oxidative stress, inducing a prothrombotic state, and aggravating endothelial dysfunction, and ultimately leads to impaired coronary flow, increased infarct size, and poor cardiac function (27, 31–33).

SIH was characterized using ABG in prior studies, defined as the random blood glucose measured within 24 hours after hospital admission, and has been demonstrated as an independent predictor of unfavorable prognosis and incorporated into some established risk scores in STEMI patients (7–9, 34). However, the ABG values were influenced by DM status, pre-admission glucose-lowering drugs, and meal timing, and the HORIZONS-AMI trial revealed that admission hyperglycemia was more predictive of mortality in STEMI patients treated with PCI without diabetes than their diabetes counterparts (35), highlighting the importance of considering DM status when interpreting ABG results, and the recorded high ABG levels do not necessarily indicate an elevated SIH. The clinical significance of elevated ABG remains controversial; whether it is merely a manifestation of the critical disease or contributes to adverse clinical outcomes still needs to be validated. What’s more, the threshold level of ABG was discordant among different studies, thereby many widely applied scoring systems, such as the GRACE score and TIMI risk score, did not incorporate ABG in the risk assessment. Therefore, the SHR was introduced as a novel indicator of SIH to adjust the impact of chronic glucose levels on HbA1c (11) and was shown to have a better prediction ability of poor prognosis than ABG (14, 15).

Cardiogenic shock is a low cardiac output state that leads to life-threatening end-organ underperfusion and hypoxia and occurs in 5-15% of STEMI patients (1–3). Despite advances in management over the past decades, the mortality of STEMI patients with CS remains unacceptably high, particularly those who developed CS during hospitalization experienced an even worse prognosis (1, 2, 4). Early identification of high-risk patients for this life-threatening complication is therefore critical to make adequate treatment decisions and improve prognosis. Prior studies have evaluated the risk factors, and several risk assessment tools, such as the IABP-SHOCK II risk score (8) and the Cardiogenic Shock Score (7), have been constructed to predict mortality in patients who have been diagnosed with CS. Nonetheless, limited studies have focused on evaluating predictors of IHCS or validating risk scores to identify patients at high risk of STEMI-related IHCS. The established models, such as the ORBI risk score (36) and machine learning algorithm (37), incorporate predictors that are either only available post-intervention (e.g., post-PCI TIMI flow and culprit lesion of the left main) (36) or are not routinely measured in clinical practice, which limits their utility in providing early discrimination. Identifying and validating practicable indicators is of great significance in adding adaptability and providing adequate information in management facilitation, therefore increasing chances of improving survival. Notably, STEMI patients with complicated CS represented higher ABG levels and were more likely combined with diabetes (1), what’s more, the higher glucose level was ranked as the top input feature and recognized as a powerful predictor for predicting CS (37), and admission glycemia > 10 mmol/L (OR: 2055, 95%CI: 1.92-3.39) was identified as a predictor of IHCS (36). However, most prior studies failed to account for glucose metabolic status and might potentially confound the interpretation of hyperglycemia’s prognostic impact.

Various clinical studies have demonstrated the prognostic significance of elevated SHR and manifested SHR as a valuable prognostic indicator. The elevated SHR has manifested significant associations with increased 30-day (HR: 1.059, 95% CI: 1.040–1.078) and 360-day all-cause mortality (HR: 1.043, 95% CI: 1.026–1.061) in patients with AMI-related CS (22). Interestingly, a significantly *U*-shaped relationship was observed between SHR and ICU mortality in critically ill patients with CS (HR: 1.511, 95%CI: 1.124-2.030) (21). Wei Xu et al. (14) confirmed an independent relationship between SHR and the risk of 30-day MACEs (HR: 1.416, 95%CI: 1.265-1.584) and all-cause death (HR: 1.507, 95%CI: 1.253-1.911) in STEMI patients. Furthermore, incorporating SHR might improve the predictive efficiency of the TIMI risk score (ΔAUC: 0.009, *P* < 0.05). Jie Yang et al. (38) revealed a J-shaped association between SHR and in-hospital cardiac death and MI (HR: 1.82, 95% CI: 1.13–2.94) after 2-year follow-up in ACS patients undergoing drug-eluting stent implantation. Additionally, elevated SHR was demonstrated to be significantly correlated with left ventricular negative remodeling after STEMI (39) and independently associated with impaired cardiac function and microvascular obstruction (33). Importantly, the addition of SHR significantly improved the predictability and clinical usefulness of the GRACE score (40). What’s more, the relationship between SHR and prognosis may differ across various patient populations, SHR demonstrated a more pronounced predictive value for in-hospital mortality in female patients compared to male patients, and exhibited a more prominent predictive value in patients without diabetes (41), age and hypertension presented a potential influence on the association between SHR and stroke risk (42), and lipids were observed to partially mediate the association between elevated SHR and risk of hypertension in older adults (43). Despite these findings, SHR as a candidate predictor for predicting IHCS has never been individually evaluated. To the best of our knowledge, the current study represents the first comprehensive evaluation of the relationship between SHR and IHCS in STEMI patients using a real-world database from population-based registries.

Several studies have reported that cardiogenic shock was more predominant in STEMI patients presented with stress hyperglycemia (14.4% vs 5.1%, plasma glucose levels > 140 mg/dL at any given time during hospitalization) (44), and STEMI patients with higher acute-to-chronic glycemic ratio had a higher incidence of cardiogenic shock regardless of DM status (45). Elsewhere, elderly AMI patients with higher SHR (≥1.25) expressed higher proportions of cardiogenic shock in the composite outcomes (25.56% vs 9.13%) (20), and the risk of cardiogenic shock was 2.47-fold in the high SHR group compared with the low SHR group in the aggregated results of meta-analysis (13). Notably, direct studies concerning the effect of the SHR on the occurrence of IHCS remain scarce. Our study for the first time illustrated that STEMI patients with higher SHR were more likely to develop IHCS during hospitalization, and the linear dose-response relationship indicated a significantly increased IHCS risk when the SHR was elevated, regardless of DM status. The elevated SHR is a powerful predictor of IHCS in patients with STEMI, particularly in patients without diabetes. The TIMI risk score and the GRACE score are the traditional tools widely utilized to predict clinical outcomes of STEMI patients, and the DM history is incorporated in the TIMI risk score, while their performance for IHCS prediction remains suboptimal. Our findings reveal that STEMI patients who developed IHCS presented with higher TIMI risk score and GRACE score on admission. More importantly, although improved predictive efficiency for MACE and mortality were reported in previous studies (14, 40), studies regarding the additive impact of the SHR on the predictive ability of the traditional scores for IHCS are limited and warranted. Our results demonstrate a significant incremental predictive performance when SHR was incorporated into traditional scores, especially in STEMI patients without diabetes. The attenuated results among STEMI patients with diabetes may be due to the insufficient sample size of patients with diabetes. Moreover, in patients with diabetes, chronic hyperglycemia impairs the body’s responsiveness to insulin and reduces sensitivity to acute glucose variations. Conversely, in non-diabetes patients, a sudden short-term spike in blood glucose can markedly exacerbate oxidative stress and endothelial dysfunction, intensify the systemic inflammatory response, and may increase the risk of thrombosis by impacting the fibrinolytic system, and consequently increase the probability of cardiovascular events. The underlying pathophysiology of the SHR with IHCS development in STEMI patients remains uncertain and might be related to insulin resistance, which has been shown to significantly affect contractile function and further decrease cardiac function (46). Additionally, stress hyperglycemia reduces endothelial nitric oxide levels, leading to insufficient organs perfusion due to vasoconstriction, which further impairs microcirculation. Lastly, hyperglycemia is associated with inflammation, oxidative stress, microvascular injury, and a prothrombotic state, all of which can result in compromised myocardial blood flow and diminished cardiac function (47).

To the best of our knowledge, there are few established clinical scoring systems for assessing the risk of IHCS in patients with STEMI, such as the ORBI risk score (36), the machine learning algorithm (37), and the EARLY SHOCK Score (48); none of the established scoring systems incorporates SHR as a predictor. Our results revealed that, although the improvement of AUC was nonsignificant by combining SHR with the classical scores, the model performance indicators focusing on the reclassification ability and the calibration of predicted probabilities, such as NRI and IDI, were significantly improved, which indicates that the incorporation of SHR provides substantive enhancements in classification accuracy or risk probability estimation, and refines the model’s utility for clinical decision-making. In clinical practice, physicians might assess the risk of IHCS using the existing scoring system, and further calculate SHR, and an elevated SHR indicates a further increased risk of IHCS based on the risk classes of the established scoring system. What’s more, further studies could explore incorporating SHR into the established scoring systems as a conventional predictor, which might facilitate the better application of SHR in clinical risk assessment processes. Generally, our study provides a new perspective for predicting IHCS incidence in STEMI patients.

Furthermore, although the SHR was recognized as a valuable prognostic marker, short-term hyperglycemia during critical illness is common and might represent an evolutionarily conserved adaptive response (27), and this physiological mechanism is beneficial in improving survival prospects. Previous studies grouped patients using tertiles (14, 16), quartiles (21), or quintiles (38), and the optimal threshold for elevated SHR was also inconsistent among different studies, especially between patients with and without DM (16). The cutoff value was usually determined by the sensitivity and specificity features of the studied index on the predicting outcomes, whereas the predictive value of an indicator varies for different outcomes, and might show varying AUC, sensitivity, and specificity in different study populations and outcomes. Despite the AUC of SHR for predicting IHCS being relatively small in this study, which might be mainly caused by the special features of SHR on IHCS identification, the value is comparable to previous studies among STEMI patients (14, 18). The AUC of SHR for predicting pulmonary infection during hospitalization was 0.624, 0.595, and 0.637 for the total patients with STEMI, DM, and non-DM patients (18); the TIMI risk score combined with SHR for predicting MACEs in STEMI patients observed an AUC of 0.773, and the AUC was 0.780 for predicting all−cause deaths (14). The AUC of SHR to predict IHCS in this study falls within the normal interval for the STEMI patients. The optimal cutoff value of SHR varies for different outcomes in different study populations, which ranged from 0.99 to 1.68 in previous studies, and showed differences in patients with and without DM (12), the optimal cut-off values of SHR for predicting MACEs in STEMI patients was 1.329 (14), and the cut-off values of 1.17, 1.3, and 1.32 were used for acute myocardial infarction patient grouping (12), as for predicting pulmonary infection in patients with STEMI, the best cut-off value was 1.073 (18). The cutoff value of SHR identified in this study is at a normal interval and broadly in line with the published studies. Determining the optimal cutoff value is crucial for implementing the corresponding clinical treatment; hence, the authoritative cutoff value of SHR still needs to be confirmed in further large-sample studies. Furthermore, in the context of an emerging protective role of sodium-glucose cotransporter-2 inhibitors (SGLT2-I) and glucagon-like peptide-1 receptor agonists (GLP-1RAs), the therapeutic approaches targeting the management of SIH need to be investigated, thereby reducing hyperglycemia and improving the prognosis.

### 4.1 Limitations

This is the first study focused on the relationship between SHR and IHCS, as well as the incremental effect and clinical usefulness of SHR on traditional risk scores in STEMI patients. Moreover, we evaluated the correlations and predictive value using a rigorously quality-controlled real-world database from a prospective, multicenter, and population-based registry. However, this study has several limitations. Firstly, only 21% of the participants were diagnosed with diabetes, the nonsignificant association observed in this study between SHR and IHCS in STEMI patients with diabetes might be due to the relatively small sample size and potential lack of statistical power to detect a statistically significant association; meanwhile, the sample size was also relatively small in the subgroup analyses, and the findings of the subgroup analyses may have been statistically underpowered and should be interpreted as exploratory based on clinical importance. Secondly, the possibility of selection bias could not be excluded, for more than half the patients in the registry were excluded from this analysis due to data deficiency; the residual confounding and the possibility of bias cannot be excluded, and the cross-sectional study design prevents the determination of a causal relationship between SHR and IHCS. Thirdly, the incremental predictive value of SHR incorporated in the scores specific for predicting IHCS (e.g., the ORBI risk score) was not evaluated due to the granularity of available data, as the registry was designed to investigate all the patients admitted with STEMI, and the post-intervention variables (e.g., post-PCI TIMI flow) were not available for patients who received fibrinolysis or conservative therapy. Fourthly, the lack of data regarding the implementation of glucose-lowering interventions during hospitalization precluded the evaluation of their effects on the prevention of IHCS incidence in STEM patients. Fifth, the type and dosage of pre-admission glucose-lowering drugs were not collected in this registry, which inhibits the evaluation of their modulatory effect on SHR’s predictive capacity in STEMI patients with DM. The SHR is the ratio of glucose and HbA1c and has the capacity to normalize the critical illness-related blood glucose increase on top of the background glycemic status; the pre-admission glucose-lowering drugs may concurrently affect the HbA1c and glucose, and might have a relatively minor impact on SHR’s predictive capacity. Sixth, the AUC of SHR for predicting IHCS in our study is relatively small; however, the AUC and cutoff value are broadly consistent with previous studies in patients with STEMI, which might be the special features of SHR on IHCS identification and have a slight impact on cutoff value accuracy. Seventh, the current analysis was mainly based on a binary approach, and the optimal cutoff values of SHR for determining SIH should be explored in further studies. Finally, all the patients included in this study were enrolled from China, with only East Asian participants; the generalizability and reproducibility of the study findings to other ethnic groups warrant further investigations to confirm the robustness.

## 5 Conclusions

SHR was significantly and independently associated with IHCS incidence and demonstrated comparable importance for predicting IHCS compared with the established risk factor in patients with STEMI, particularly in STEMI patients without diabetes. The addition of SHR to the traditional risk scores manifested significant incremental predictive performance. Higher SHR should be considered a valuable indicator and can be incorporated into future models for predicting IHCS. Large-scale studies are still needed to evaluate its predictive effect in patients with diabetes, and the optimal cutoff value also needs to be verified in further studies.

## Data Availability

The data supporting this study are included in the article and supplementary materials. The datasets analyzed during the current study are not publicly available due to ethical issues, but de-identified data are available from the corresponding author with permission of the IRB upon reasonable request.
