# Supplementary material for: Impact of stress hyperglycemia ratio on incidence of in-hospital cardiogenic shock in patients with ST-elevation myocardial infarction: a prospective, multicenter study
Source: Front Endocrinol (Lausanne). 2025 Dec 12;16:1677084. doi: 10.3389/fendo.2025.1677084 (PMC12740882; doi:10.3389/fendo.2025.1677084)
Supplement: Supplementary file 1 [file DataSheet1.docx]

**Supplement materials**

**Table of content**

| **Serial number** | **Legend** | **Page** |
| --- | --- | --- |
| *Supplement figure 1* | *Flow chart of STEMI patients’ selection and grouping.* | 1 |
| *Supplement figure 2* | *The ROC curve of SHR for the occurrence of IHCS in STEMI patients.* | 2 |
| *Supplement figure 3* | *The incidence of IHCS among the two groups as determined using SHR in STEMI patients according to diabetes status* | 5 |
| *Supplement figure 4* | *The permutation feature importance analysis of SHR and the established risk factors for the predicting of IHCS.* | 6 |
| *Supplement table 1* | *List of hospitals in the Henan STEMI registry.* | 8 |
| *Supplement table 2* | *The performance of the TIMI risk score, GRACE score, and SHR on the incidence of IHCS estimated by the internal bootstrap validation method.* | 10 |
| *Supplement table 3* | *Established risk factors associated with prognosis and included in the TIMI risk score and GRACE score for STEMI patients.* | 12 |
| *Supplement table 4* | *Subgroup analysis for the association of SHR with IHCS.* | 13 |

**Supplement figure 1.** Flow chart of STEMI patients’ selection and grouping. ST-segment elevation myocardial infarction; SHR, stress hyperglycemia ratio; DM, diabetes mellitus.

**
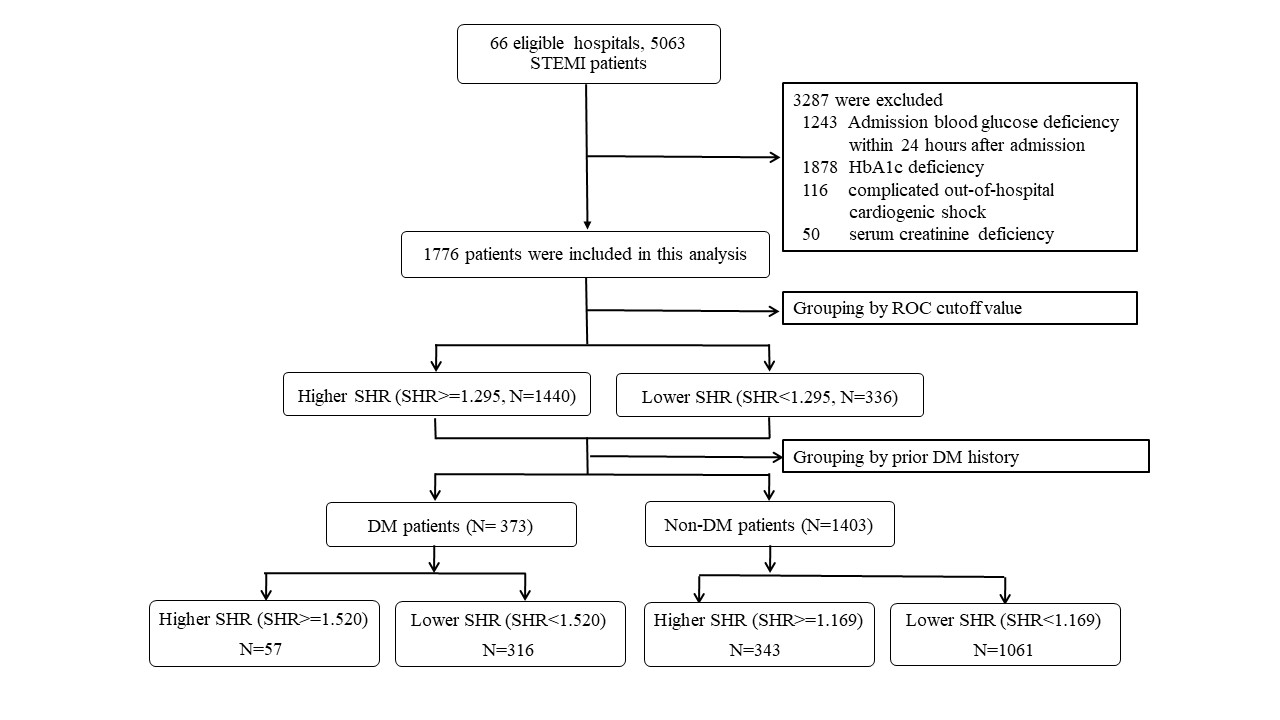
**

**Supplement figure 2.** The ROC curve of SHR for the occurrence of IHCS in STEMI patients. ST-segment elevation myocardial infarction; SHR, stress hyperglycemia ratio; DM, diabetes mellitus; IHCS, in-hospital cardiogenic shock; ROC curve, receiver operator characteristic curve.

(A) The ROC of SHR for IHCS among the total patients.

**
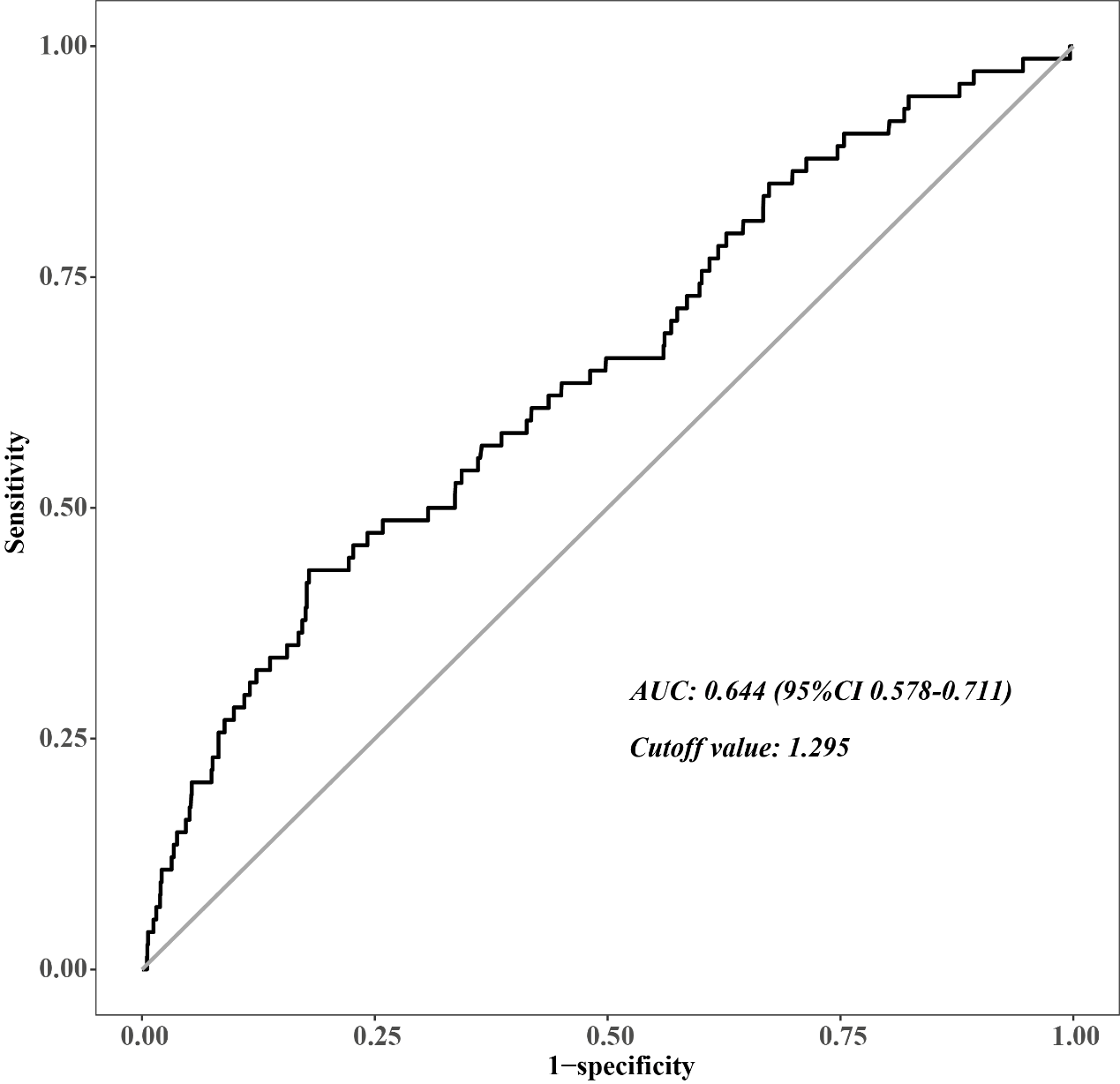
**

(B) The ROC of SHR for IHCS among DM patients

**
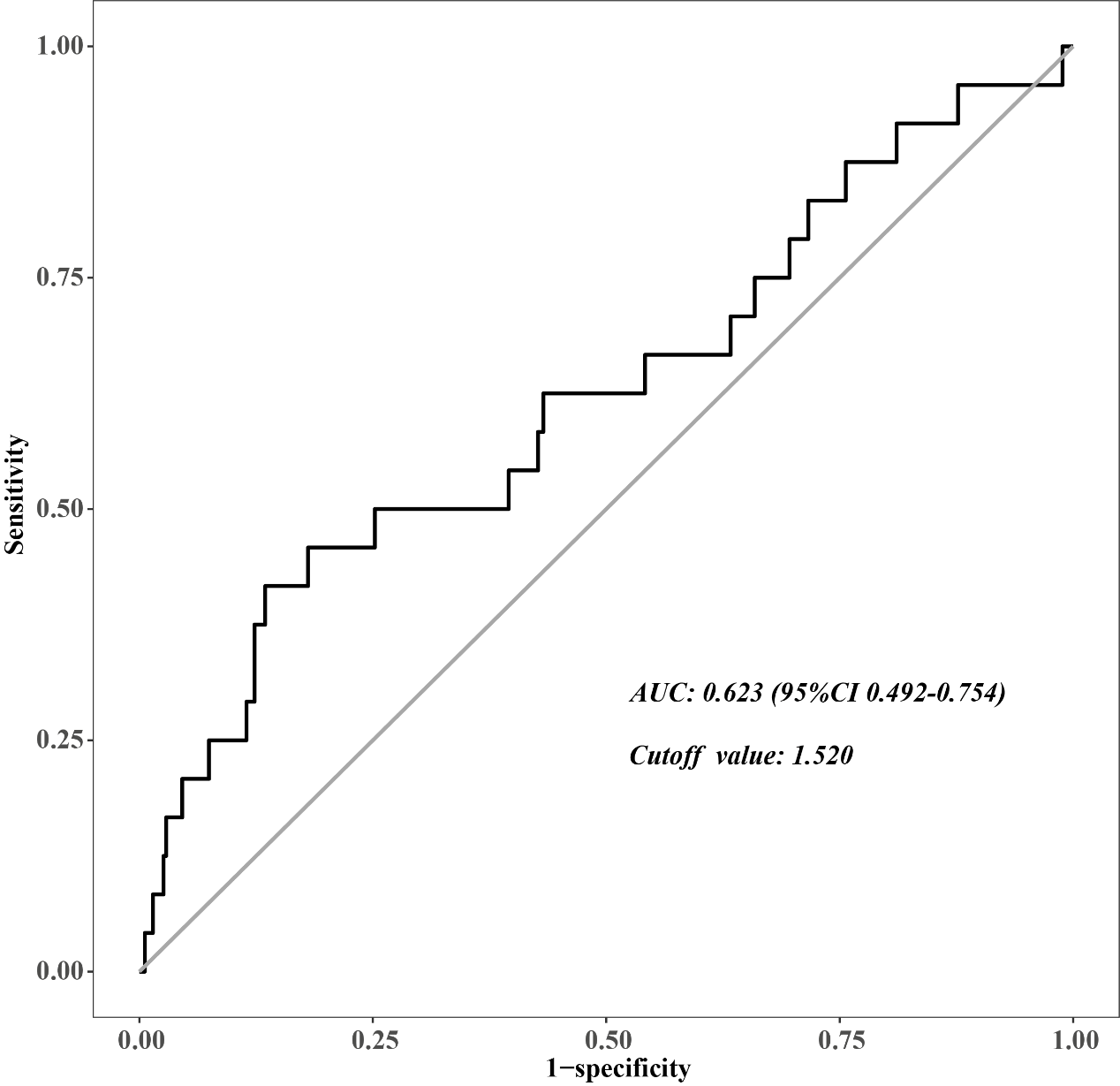
**

(C) The ROC of SHR for IHCS among Non-DM patients

**
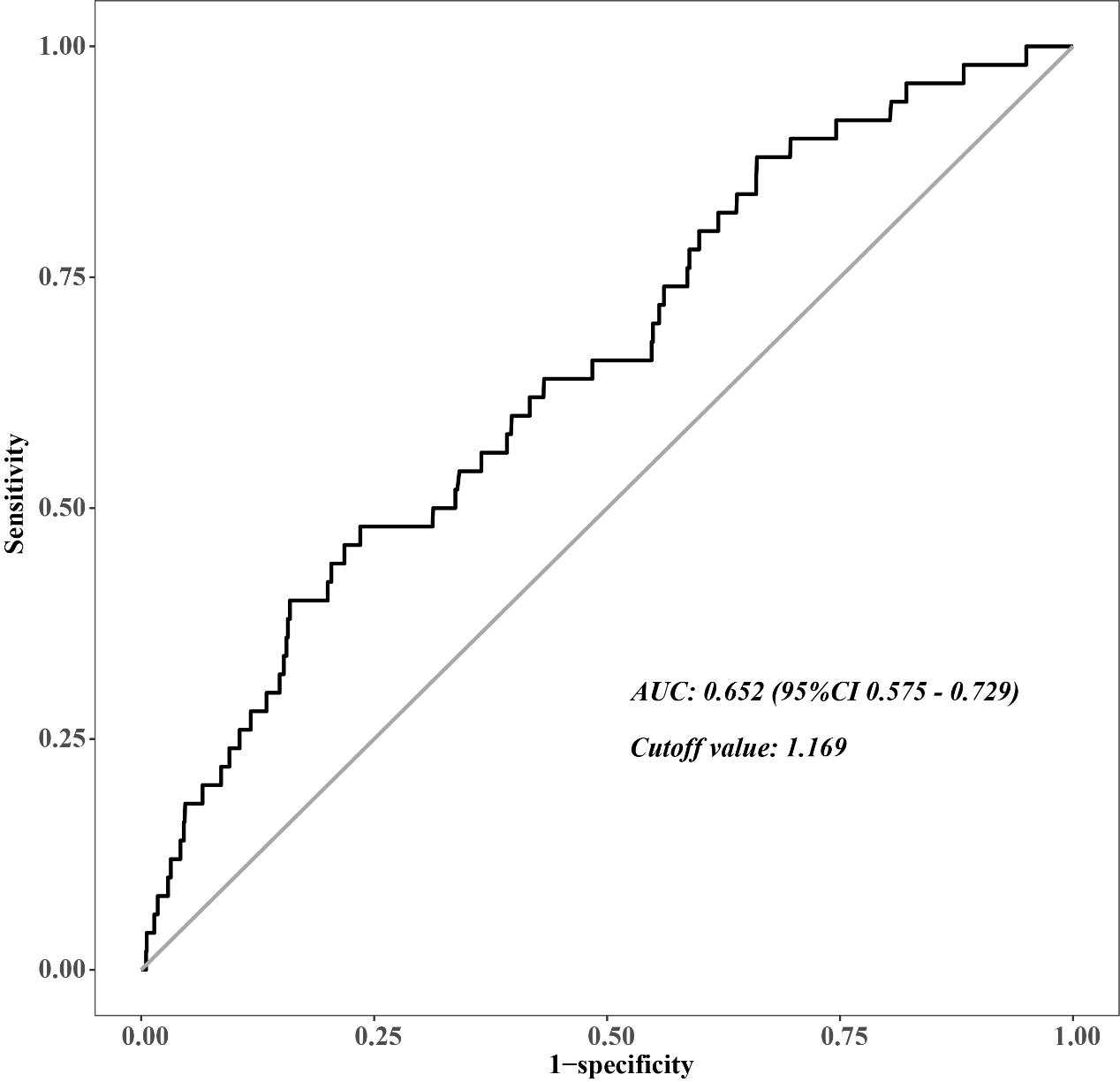
**

**Supplement figure 3.** The incidence of IHCS among the two groups as determined using SHR in STEMI patients according to diabetes status.

**
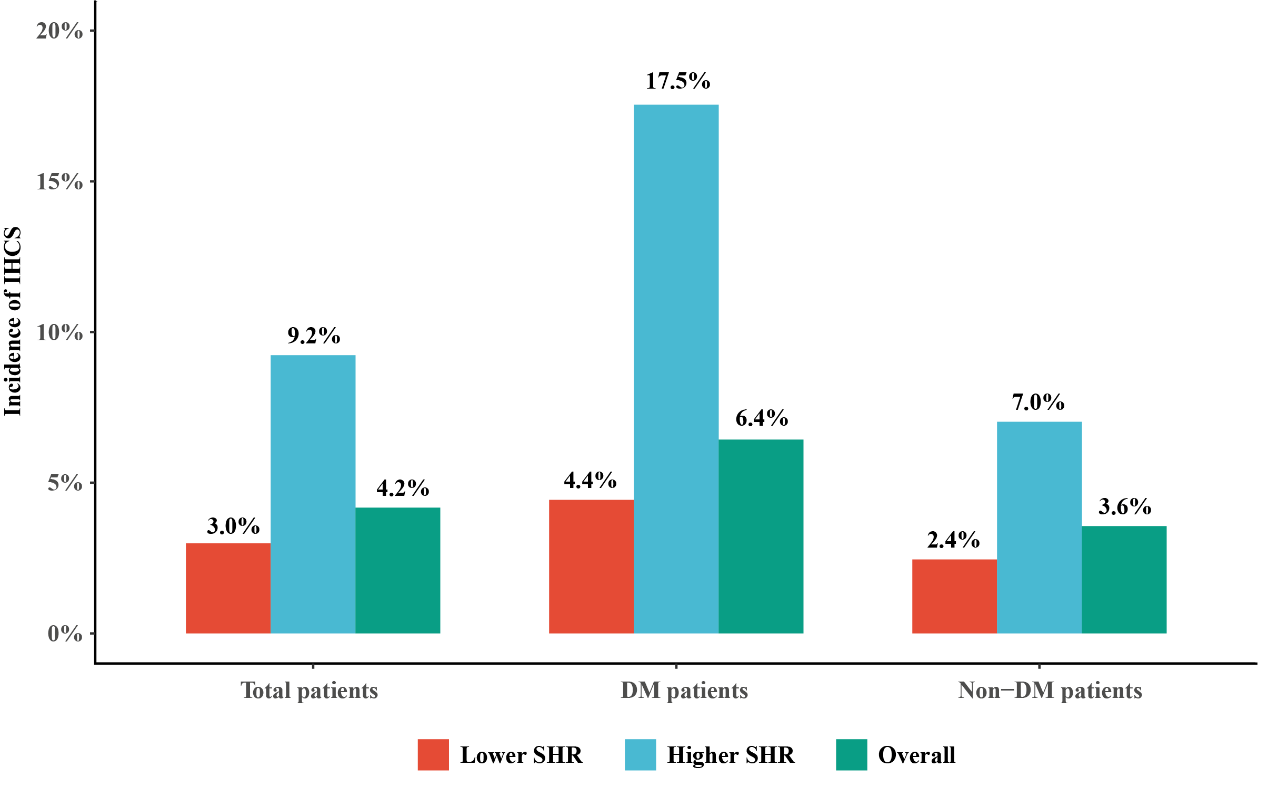
**

**Supplement figure 4.** The permutation feature importance analysis of SHR and the established risk factors for the predicting of IHCS.

(A) The total patients


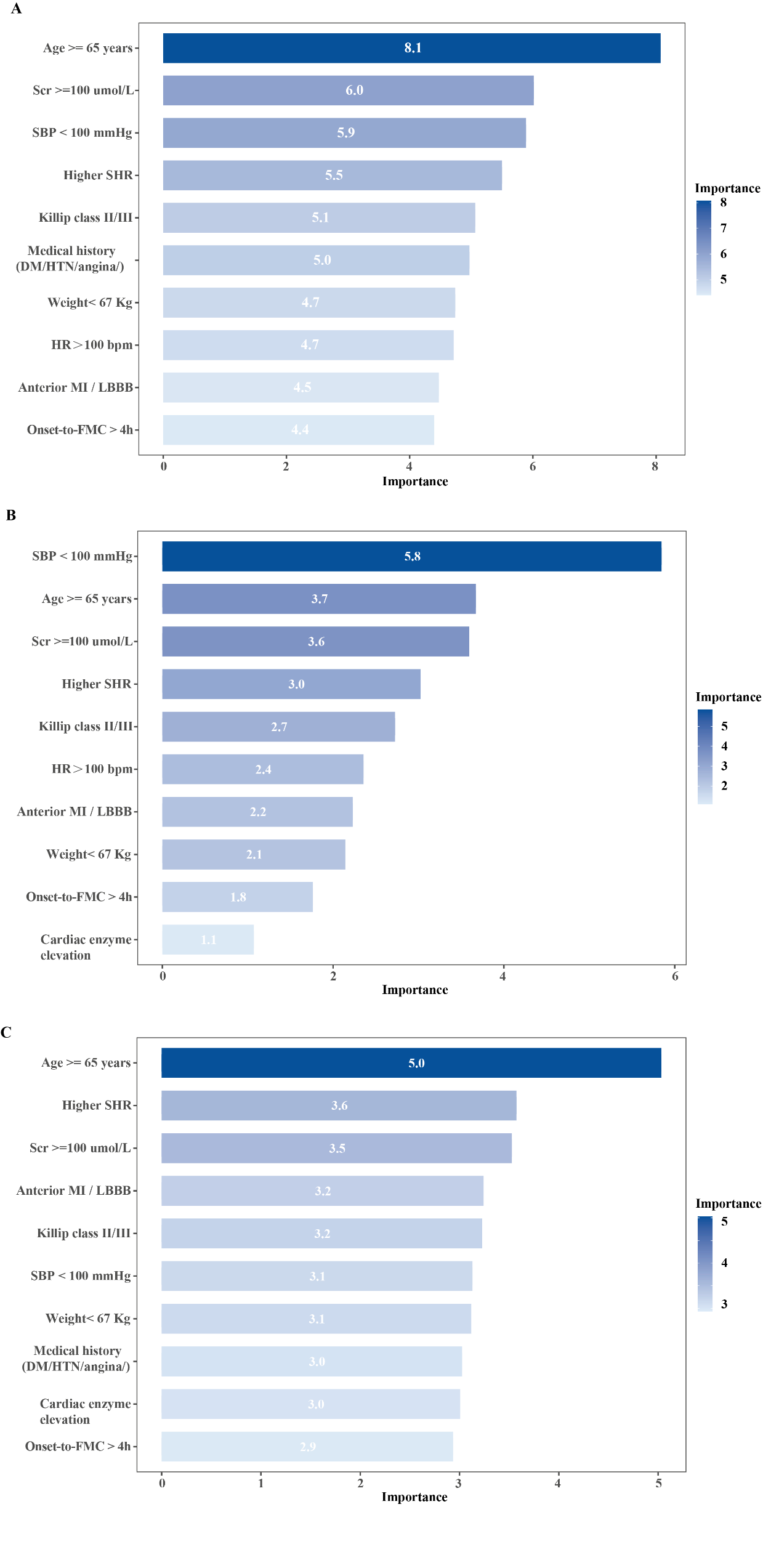


(B) Patients with diabetes


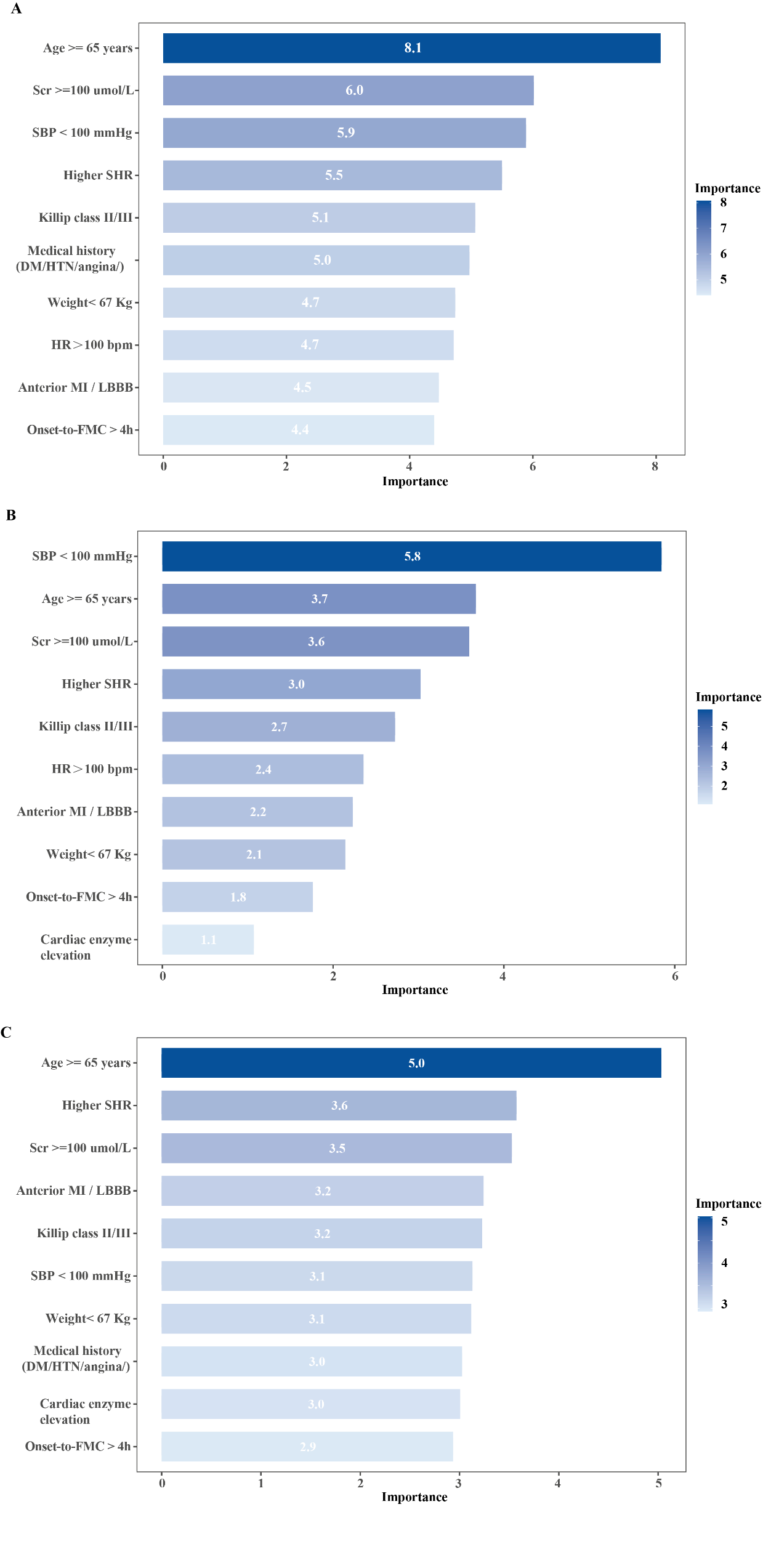


(C) Patients without diabetes.


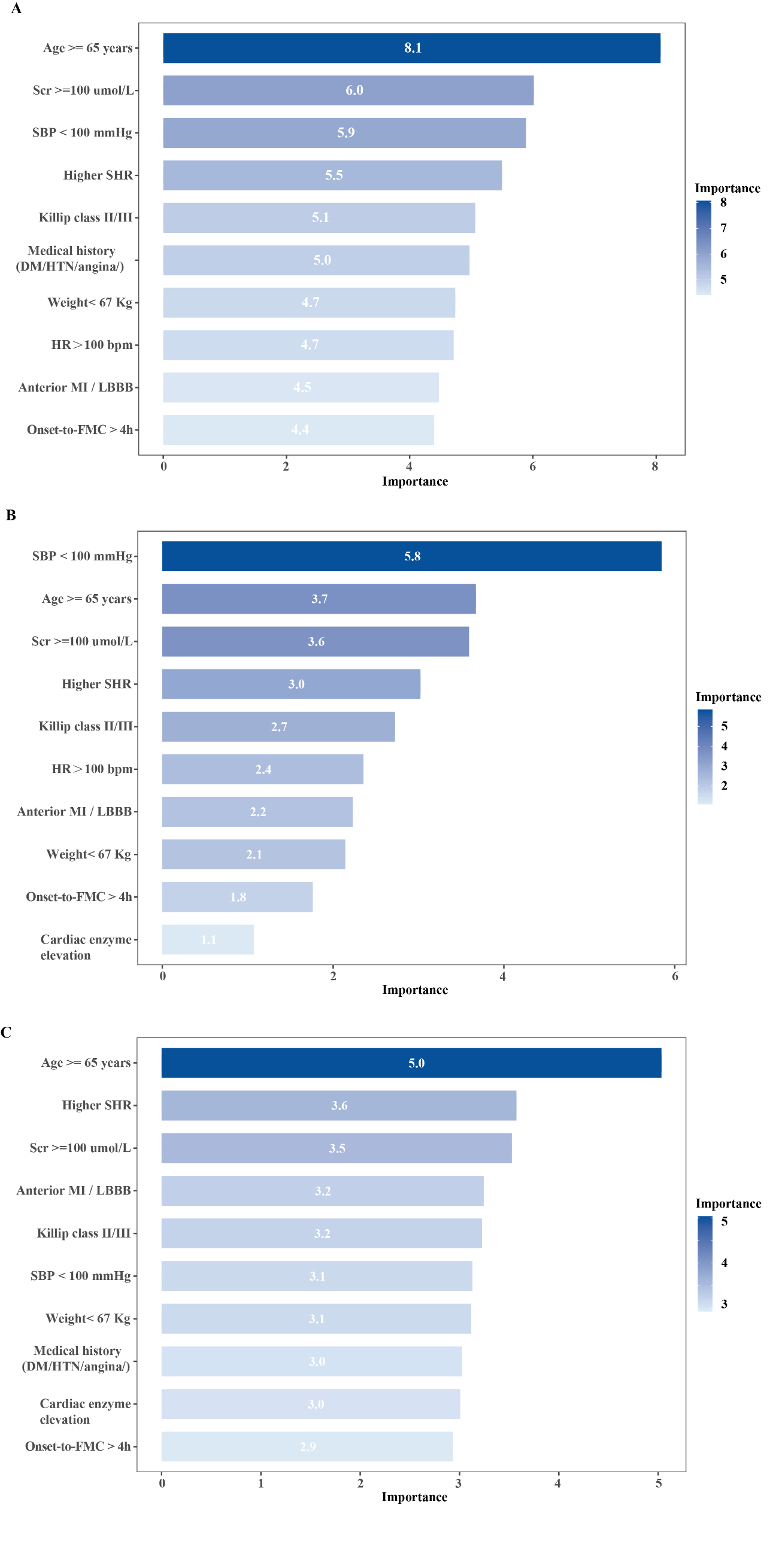


**Supplement table 1.** List of hospitals in the *Henan STEMI registry*.

| **NO** | **Hospital** | **Prefecture/City** | **Grade** |
| --- | --- | --- | --- |
| 1 | Zhengzhou University People's Hospital | Zhengzhou | Tertiary |
| 2 | Xinxiang Central Hospital | Xinxiang | Tertiary |
| 3 | The First Affiliated Hospital of Henan Science and Technology University | Luoyang | Tertiary |
| 4 | The First People's Hospital of Shangqiu | Shangqiu | Tertiary |
| 5 | Zhumadian central hospital | Zhumadian | Tertiary |
| 6 | The Third Affiliated Hospital of Xinxiang medical college | Xinxiang | Tertiary |
| 7 | The Second People's Hospital of Xinxiang | Xinxiang | Tertiary |
| 8 | The Second People's Hospital of Nanyang | Nanyang | Tertiary |
| 9 | The People's Hospital of Jiaozuo | Jiaozuo | Tertiary |
| 10 | Puyang Oil Field General Hospital | Puyang | Tertiary |
| 11 | The First People's Hospital of Xinxiang | Xinxiang | Tertiary |
| 12 | The People's Hospital of Hebi | Hebi | Tertiary |
| 13 | The Second People's Hospital of Pingdingshan | Pingdingshan | Tertiary |
| 14 | The First Affiliated Hospital of Henan University | Kaifeng | Tertiary |
| 15 | Nanshi Hospital of Nanyang | Nanyang | Tertiary |
| 16 | The Third People's Hospital of Luoyang | Luoyang | Tertiary |
| 17 | Xiping County People's Hospital | Zhumadian | Secondary |
| 18 | The People's Hospital of Yongcheng | Shangqiu | Secondary |
| 19 | Xihua County People's Hospital | Zhoukou | Secondary |
| 20 | Shangcai County People's Hospital | Zhumadian | Secondary |
| 21 | The People's Hospital of Gongyi | Zhengzhou | Secondary |
| 22 | The First People's Hospital of Lingbao | Sanmenxia | Secondary |
| 23 | The People's Hospital of Xingyang | Zhengzhou | Secondary |
| 24 | Ningling County People's Hospital | Shangqiu | Secondary |
| 25 | Fengqiu County People's Hospital | Xinxiang | Secondary |
| 26 | New area People's Hospital of Luoyang | Luoyang | Secondary |
| 27 | Mianchi County People's Hospital | Sanmenxia | Secondary |
| 28 | Wen County People's Hospital | Jiaozuo | Secondary |
| 29 | Pingyu County People's Hospital | Zhumadian | Secondary |
| 30 | Xiayi County People's Hospital | Shangqiu | Secondary |
| 31 | The People's Hospital of Qinyang | Jiaozuo | Secondary |
| 32 | Tongxu County hospital of Chinese medicine | Kaifeng | Secondary |
| 33 | Song County People's Hospital | Luoyang | Secondary |
| 34 | The Third People's Hospital of Shangqiu | Shangqiu | Secondary |
| 35 | Zhecheng County People's Hospital | Shangqiu | Secondary |
| 36 | The Second People's Hospital of Jiyuan | Jiyuan | Secondary |
| 37 | The Second People's Hospital of Mengjin County | Luoyang | Secondary |
| 38 | Yudong Hospital of the First Affiliated Hospital of Henan University of Chinese Medicine | Shangqiu | Secondary |
| 39 | The People's Hospital of Yanshi | Luoyang | Secondary |
| 40 | Xuchang County People's Hospital | Xuchang | Secondary |
| 41 | Neixiang County People's Hospital | Nanyang | Secondary |
| 42 | The People's Hospital of Dengfeng | Zhengzhou | Secondary |
| 43 | Nanzhao County People's Hospital | Nanyang | Secondary |
| 44 | Minquan County People's Hospital | Shangqiu | Secondary |
| 45 | Yuhzou City hospital of Chinese Medicine | Xuchang | Secondary |
| 46 | Tongxu County People's Hospital | Kaifeng | Secondary |
| 47 | The Second People's Hospital of Xiayi County | Shangqiu | Secondary |
| 48 | The Sixteenth People's Hospital of Zhengzhou | Zhengzhou | Secondary |
| 49 | Zhecheng County hospital of Chinese Medicine | Shangqiu | Secondary |
| 50 | Sheqi County People's Hospital | Nanyang | Secondary |
| 51 | The People's Hospital of Wugang | Pingdingshan | Secondary |
| 52 | Xinye County People's Hospital | Nanyang | Secondary |
| 53 | Weishi County People's Hospital | Kaifeng | Secondary |
| 54 | Runan County People's Hospital | Zhumadian | Secondary |
| 55 | Yichuan County hospital of Chinese Medicine | Luoyang | Secondary |
| 56 | Yucheng County People's Hospital | Shangqiu | Secondary |
| 57 | Minquan County hospital of Chinese Medicine | Shangqiu | Secondary |
| 58 | The People's Hospital of Jiaozuo Macun District | Jiaozuo | Secondary |
| 59 | Qi County People's Hospital | Kaifeng | Secondary |
| 60 | Ye County People's Hospital | Pingdingshan | Secondary |
| 61 | Huangchuan County People's Hospital | Xinyang | Secondary |
| 62 | Queshan County People's Hospital | Zhumadian | Secondary |
| 63 | The Central Hospital of Yima Coal Industry Group CO. LTD | Sanmenxia | Secondary |
| 64 | Suiping County People's Hospital | Zhumadian | Secondary |
| 65 | Fangcheng County People's Hospital | Nanyang | Secondary |
| 66 | The Second People's Hospital of Xichuan County | Nanyang | Secondary |

**Supplement table 2. The performance of TIMI risk score, GRACE score, and SHR on incidence of IHCS estimated by internal bootstrap validation method.**

|  | **AUC (95% CI)** | ***P* value** | **Optimism (*SD*)** | **Bias-corrected**  **AUC (95% CI)** | ***P* value** |
| --- | --- | --- | --- | --- | --- |
| **Total patients** |  |  |  |  |  |
| TIMI risk score | 0.737 (0.648, 0.818) | ＜0.001 | 0.001 (0.043) | 0.737 (0.648, 0.818) | ＜0.001 |
| TIMI risk score + SHR | 0.754 (0.672, 0.830) | ＜0.001 | 0.002 (0.041) | 0.755 (0.672, 0.830) | ＜0.001 |
| GRACE score | 0.746 (0.669, 0.825) | ＜0.001 | 0.002 (0.041) | 0.746 (0.669, 0.825) | ＜0.001 |
| GRACE score + SHR | 0.760 (0.688, 0.831) | ＜0.001 | 0.004 (0.039) | 0.761 (0.688, 0.831) | ＜0.001 |
| **DM patients** |  |  |  |  |  |
| TIMI risk score | 0.860 (0.746, 0.940) | ＜0.001 | 0.003 (0.049) | 0.860 (0.746, 0.940) | ＜0.001 |
| TIMI risk score + SHR | 0.859 (0.749, 0.942) | ＜0.001 | 0.009 (0.049) | 0.863 (0.749, 0.942) | ＜0.001 |
| GRACE score | 0.836 (0.720, 0.929) | ＜0.001 | 0.003 (0.053) | 0.836 (0.720, 0.929) | ＜0.001 |
| GRACE score + SHR | 0.824 (0.691, 0.930) | ＜0.001 | 0.009 (0.059) | 0.828 (0.691, 0.930) | ＜0.001 |
| **Non-DM patients** |  |  |  |  |  |
| TIMI risk score | 0.678 (0.567, 0.789) | ＜0.001 | 0.002 (0.056) | 0.678 (0.567, 0.789) | ＜0.001 |
| TIMI risk score + SHR | 0.706 (0.608, 0.809) | ＜0.001 | 0.003 (0.052) | 0.707 (0.608, 0.809) | ＜0.001 |
| GRACE score | 0.706 (0.605, 0.804) | ＜0.001 | 0.002 (0.050) | 0.706 (0.605, 0.804) | ＜0.001 |
| GRACE score + SHR | 0.730 (0.640, 0.817) | ＜0.001 | 0.003 (0.045) | 0.731 (0.640, 0.817) | ＜0.001 |

**Abbreviation:** AUC, area under the curve; TIMI, Thrombolysis In Myocardial Infarction; GRACE; Global Registry of Acute Coronary Events. SHR, stress hyperglycemia ratio; DM, diabetes mellitus.

**Supplement table 3.** Established risk factors associated with prognosis and included in the TIMI risk score and GRACE score for STEMI patients. TIMI, Thrombolysis In Myocardial Infarction; GRACE; Global Registry of Acute Coronary Events.

| ***NO*** | ***Risk factors in TIMI risk score*** | ***Risk factors in GRACE score*** |
| --- | --- | --- |
| 1 | Age 65-74 / ≥ 75 years | Age |
| 2 | Systolic Blood Pressure < 100 mmHg | Systolic Blood Pressure |
| 3 | Heart rate > 100 bpm | Heart rate |
| 4 | Killip II-IV | Killip class |
| 5 | Anterior STE or LBBB | Creatinine |
| 6 | Diabetes, h/o HTN, or h/o angina | Cardiac arrest at admission |
| 7 | Weight < 67 Kg | ST-segment deviation |
| 8 | Time to treatment > 4 hours | Elevated cardiac enzymes/markers |

**Supplement table 4.** Subgroup analysis for the association of SHR with IHCS.

| **Subgroups** | **Event rate (95% CI),%** | **OR (95% CI)** | ***P value*** | ***P-interaction*** |
| --- | --- | --- | --- | --- |
| **Age** |  |  |  |  |
| <65 years | 2.25 (1.34,3.15) | 3.19 (1.29,7.88) | 0.014 | <0.001 |
| ≥65 years | 6.78 (4.98,8.58) | 2.75 (1.44,5.26) | 0.004 |  |
| **Gender** |  |  |  |  |
| Female | 6.73 (4.36,9.09) | 1.95 (0.83,4.56) | 0.117 | 0.018 |
| Male | 3.35 (2.38,4.31) | 4.02 (2.03,7.95) | <0.001 |  |
| **Hospital grade** |  |  |  |  |
| Secondary | 2.33 (1.20,3.46) | 2.36 (0.79,7.07) | 0.120 | 0.826 |
| Tertiary | 5.32 (3.99,6.65) | 3.27 (1.69,6.33) | 0.003 |  |
| **Hypertension** |  |  |  |  |
| Yes | 5.40 (3.81,6.99) | 2.96 (1.48,5.92) | 0.004 | 0.563 |
| No | 3.21 (2.11,4.30) | 3.47 (1.53,7.89) | 0.005 |  |
| **Dyslipidemia** |  |  |  |  |
| Yes | 4.22 (3.03,5.41) | 5.51 (2.83,10.74) | <0.001 | 0.062 |
| No | 4.08 (2.60,5.56) | 1.17 (0.44,3.07) | 0.742 |  |
| **Reperfusion therapy** |  |  |  |  |
| Conservative Strategy | 4.25 (2.78,5.71) | 3.36 (1.48,7.63) | 0.006 | 0.762 |
| PCI | 4.67 (3.04,6.31) | 2.60 (1.10,6.14) | 0.032 |  |
| Fibrinolysis | 3.22 (1.50,4.94) | 5.32 (1.24,22.90) | 0.027 |  |
| **Onset-to-FMC within 4 hours** | |  |  |  |
| Yes | 4.16 (2.81,5.51) | 2.96 (1.36,6.45) | 0.009 | 0.995 |
| No | 4.17 (2.89,5.45) | 3.40(1.64,7.04) | 0.002 |  |
| **Anterior MI** |  |  |  |  |
| Yes | 4.56 (3.29,5.83) | 5.06 (2.63,9.74) | <0.001 | 0.037 |
| No | 3.62 (2.28,4.97) | 1.03 (0.35,3.06) | 0.961 |  |

**Abbreviation:** OR, odd ratio; CI, confidence intervals. FMC, first medical contact.
